# Healthcare Workers' Acceptance and Willingness to Implement a Pragmatic Triple‐Component Enhanced Recovery After Surgery Strategy (T‐ERAS): A Cross‐Sectional Study in Ethiopian Public Hospitals

**DOI:** 10.1002/wjs.70319

**Published:** 2026-03-22

**Authors:** Fitsum Kifle, Peniel Kenna, Bethelehm Muleye, Kokeb Desta, Salome Maswime, Bruce Biccard

**Affiliations:** ^1^ Global Surgery Division, Department of Surgery, Faculty of Health Sciences University of Cape Town Cape Town South Africa; ^2^ Network for Perioperative and Critical Care Debre Berhan University, Asrat Woldyes Health Sciences Campus Debre Berhan Ethiopia; ^3^ Network for Perioperative and Critical Care Global Partners for Improving Surgical System Addis Ababa Ethiopia; ^4^ Department of Quality and Health Management Information System Kidus Peteros Hospital Addis Ababa Ethiopia; ^5^ Department of Anaesthesia and Perioperative Medicine Groote Schuur Hospital, University of Cape Town Cape Town Western Cape South Africa; ^6^ Department of Clinical Neurosciences University of Oxford Oxford UK

**Keywords:** ERAS, healthcare provider perspectives, implementation barriers, low‐resource settings

## Abstract

**Background:**

Enhanced recovery after surgery (ERAS) protocols have demonstrated substantial benefits in improving postoperative outcomes. However, in low‐resource settings such as Ethiopia, ERAS adoption remains limited, necessitating pragmatic context‐sensitive implementation approaches. The perspectives of frontline perioperative providers who are central to implementation have rarely been systematically assessed. This study aimed to evaluate acceptance, perceived benefits, implementation challenges, and readiness for sustained adoption of a pragmatic triple enhanced recovery after surgery (T‐ERAS) protocol, comprising early oral intake, early ambulation, and early urinary catheter removal, among healthcare workers participating in a multisite ERAS trial in Ethiopia.

**Methods:**

A descriptive cross‐sectional survey was conducted among 20 perioperative care providers, including surgeons, obstetricians, anesthetists, and nurses, from five public hospitals involved in the ERAS implementation trial. A structured questionnaire administered via Google Forms captured demographic characteristics, ERAS knowledge and exposure, perceived benefits and challenges, and willingness to adopt ERAS practices. Data were analyzed using descriptive statistics and thematic summaries.

**Results:**

Twenty perioperative care providers participated, of whom 13 (65.0%) were male, and 15 (75.0%) were affiliated with tertiary‐level public hospitals. Twelve respondents (60.0%) reported being very familiar with ERAS protocols, although only 11 (55.0%) had directly participated in implementation. Sixteen participants (80.0%) believed ERAS improves patient outcomes; however, adherence varied, with 8 (42.1%) reporting rarely applying the protocol. Key implementation challenges included limited resources (85.0%), insufficient staff training (70.0%), resistance to change (50.0%), and inadequate patient education (50.0%). Despite these barriers, 17 participants (85.0%) expressed willingness to adopt ERAS practices permanently, and all (100.0%) were open to further training. The T‐ERAS components were viewed favorably, with 15 participants (75.0%) rating each as effective.

**Conclusion:**

This study demonstrates high awareness and willingness among perioperative professionals in Ethiopian public hospitals to adopt ERAS principles, while highlighting persistent system‐level barriers to consistent implementation. Strengthening institutional support, expanding training, and promoting locally led context‐sensitive ERAS pathways such as T‐ERAS may facilitate sustainable scale‐up in low‐resource settings.

## Graphical abstract

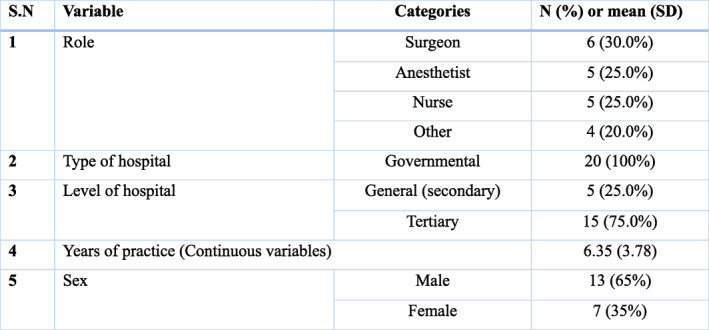

## 1 Background

Enhanced recovery after surgery (ERAS) protocols have demonstrated substantial benefits in improving postoperative outcomes and reducing hospital length of stay. However, the successful implementation of ERAS depends not only on the availability of protocols but also on healthcare workers' acceptance, beliefs, and willingness to adopt these practices, which frequently represent key barriers to sustained implementation [1]. Understanding the attitudes and perceptions of frontline providers is, therefore, critical for effective ERAS adoption.

In high‐income settings, ERAS has become standard practice across multiple surgical specialties, supported by strong evidence, institutional commitment, and well‐resourced multidisciplinary perioperative teams [2, 3, 4]. Even in these contexts, however, implementation challenges persist, including resistance to change, limited multidisciplinary coordination, and skepticism toward protocol‐driven care [5, 6, 7]. These findings highlight the importance of “human factors” in ERAS success, underscoring that implementation is influenced as much by provider engagement as by clinical evidence.

Implementing ERAS in resource‐limited settings introduces additional complexity. Studies from transitional health systems in Latin America and Southeast Asia report that although healthcare providers often support the ERAS principles conceptually, institutional, cultural, and logistical constraints frequently limit full implementation [6, 8, 9]. Reported barriers include gaps in clinical training, inconsistent adherence to protocols, limited interdisciplinary collaboration, and administrative bottlenecks. In Africa, evidence remains sparse and largely confined to single‐center studies from a small number of countries; nevertheless, available data similarly indicate that resistance to change and system‐level constraints hinder widespread ERAS adoption [10].

Given these challenges, implementing the full ERAS pathway may not be feasible in many low‐resource contexts, making it essential to identify high‐impact postoperative ERAS components that can serve as pragmatic entry points for adoption. Prior to initiating the intervention, a retrospective analysis identified five postoperative ERAS elements associated with improved surgical outcomes [11]. Based on this evidence, a pragmatic three‐component postoperative ERAS strategy, hereafter referred to as pragmatic triple ERAS (T‐ERAS), was developed and implemented in a prior multicentre trial, where it was associated with reductions in postoperative hospital length of stay by approximately 8 h and in postoperative complications by 2.5%, respectively [12].

In this study, we aimed to assess the perspectives of frontline perioperative providers, surgeons, obstetricians, anesthetists, nurses, and other key personnel, directly involved in implementing the T‐ERAS strategy in their facilities as part of a broader national surgical quality initiative in Ethiopia. Specifically, we explored providers' acceptance of T‐ERAS and their willingness to continue implementing it in routine surgical care. By focusing on the experiences of those delivering care on the ground, this study seeks to generate actionable insights to inform national surgical policy and support the development of scalable context‐sensitive ERAS pathways in resource‐constrained settings.

## 2 Method

### 2.1 Study Design and Setting

This study used a descriptive cross‐sectional survey design to assess perioperative care providers' knowledge, perceptions, and acceptance of ERAS protocols following the implementation of T‐ERAS at five public hospitals in Ethiopia between March and April 2025. These hospitals were part of a multisite ERAS trial and represented a mix of tertiary and general care levels. Collectively, the participating hospitals comprised more than 2000 hospital beds, including approximately 270 surgical beds and 230 obstetric beds. All hospitals had one or more post‐anesthesia care units (PACUs), with an estimated 60 PACU nurses assigned daily across sites.

Ethical approval was obtained from the University of Cape Town Faculty of Health Sciences Human Research Ethics Committee (Protocol Number: 220/2024), Debre Berhan University (Protocol Number: IRB‐095), and the Arba Minch University Institutional Research Ethics Review Board (Protocol Number: IRB/2320/2024). All participants provided informed consent electronically via Google Forms. Data were collected anonymously, and strict confidentiality was maintained throughout the study. No artificial intelligence generated content (AIGC) tools, such as ChatGPT and others based on large language models (LLMs), were used in developing any portion of this manuscript.

### 2.2 Study Population and Sampling

A total of 20 perioperative care providers were purposively selected from the five hospitals participating in the T‐ERAS trial. As part of the intervention, professionals at these sites received structured training on safe operating room (OR) practices and ERAS protocols. From this pool of trained staff, four participants were recruited per hospital, resulting in a total sample of 20 respondents.

The sample included surgeons, obstetricians, anesthetists, nurses, and other professionals directly involved in perioperative care, such as hospital quality officers; some participants held dual clinical or administrative roles. To ensure balanced representation across institutions and professional categories, one individual from each key professional group was recruited per site.

The sample size was considered appropriate for the study's descriptive and exploratory objectives, which focused on understanding implementation experiences, perceived barriers, and the acceptability of ERAS among trained implementers, rather than on estimating population parameters or testing hypotheses. Accordingly, the study was not designed or powered for inferential statistical analyses.

### 2.3 Data Collection

Data were collected using a structured self‐administered questionnaire using Google Forms. The questionnaire link was distributed via institutional email to the selected participants at each trial site. The tool consisted of multiple sections that captured demographic characteristics, professional roles, familiarity with ERAS protocols, perceived benefits and challenges, experience with the trial's triple intervention strategy (early postoperative feeding and drinking, early ambulation, and early urinary catheter removal), and willingness to adopt ERAS practices. The questionnaire included multiple‐choice, Likert‐scale, and multiple‐select questions, as shown in Supporting Information S1: Appendix 1. The participants were also given the opportunity to provide open‐ended feedback on their implementation experience.

### 2.4 Data Analysis

Data were exported to Microsoft Excel for initial cleaning and subsequently analyzed using SPSS version 24. Descriptive statistics were used to summarize the findings. Categorical variables were reported as frequencies and percentages, and continuous variables were summarized using means and standard deviations depending on the distribution. For questions that allow multiple selections (e.g., perceived benefits or support needs), each response option was converted into a binary (yes/no) variable to enable precise frequency analysis of individual items. Open‐ended responses from eight responders were thematically categorized and manually coded into meaningful groups to enable inclusion in frequency summaries. The results were organized and reported using structured tables aligned with four thematic domains: (1) demographic and professional background; (2) knowledge, exposure, and perceived benefits of ERAS; (3) implementation challenges and support needs; and (4) willingness to adopt ERAS and perceived effectiveness of the T‐ERAS intervention strategy.

## 3 Results

The characteristics of the participants are shown in Table 1. Twenty perioperative care providers participated in this study. Surgeons accounted for the largest group, 6 (30.0%), followed by anesthetists and nurses, each 5 (25%). The remaining 4 (20%) were categorized as other perioperative staff. All participants were affiliated with government hospitals, with no representation from private or NGO facilities. Most respondents were from tertiary hospitals, 15 (75%). The participants reported a mean of 6.35 (3.78) years of medical practice. 13 (65%) were male, and 7(35%) were female.

**TABLE 1:** Demographic and professional backgrounds of perioperative care providers (*n* = 20) in Ethiopia.

S/N	Variable	Categories	*N* (%) or mean (SD)
1	Role	Surgeon	6 (30.0%)
		Anesthetist	5 (25.0%)
		Nurse	5 (25.0%)
		Other	4 (20.0%)
2	Type of hospital	Governmental	20 (100%)
3	Level of hospital	General (secondary)	5 (25.0%)
		Tertiary	15 (75.0%)
4	Years of practice (continuous variables)		6.35 (3.78)
5	Sex	Male	13 (65%)
		Female	7 (35%)

Abbreviation: SD, standard deviation.

The knowledge, exposure, and perceived benefits of ERAS are shown in Table 2. Most respondents reported a high degree of familiarity with ERAS protocols: 12 (60.0%) indicated they were “very familiar,” and 1 (5.0%) was “extremely familiar.” 11 (55.0%) reported having participated in implementing one or more components of the Perioperative ERAS protocol at their respective hospitals. However, the consistency of protocol application varied considerably: from 3 (15.8%) “always” using ERAS protocols, and 3 (15.8%) “often” or “sometimes” to 8 (42.1%) reporting “rarely.”

**TABLE 2:** Knowledge, exposure, and perceived benefits of ERAS among perioperative care providers (*n* = 20) in Ethiopia.

S/N	Variable	Category	*n* (%)
1	Familiarity with ERAS	Extremely familiar	1 (5.0%)
		Very familiar	12 (60.0%)
		Moderately familiar	3 (15.0%)
		Slightly familiar	2 (10.0%)
		Not familiar	2 (10.0%)
2	Participation in implementation	Yes	11 (55.0%)
		No	9 (45.0%)
3	Frequency of ERAS practice	Always	3 (15.8%)
		Often	3 (15.8%)
		Sometimes	3 (15.8%)
		Rarely	8 (42.1%)
		Never	2 (10.5%)
4	Belief in ERAS benefits	Strongly agree	9 (45.0%)
		Agree	7 (35.0%)
		Neutral	2 (10.0%)
		Disagree	0 (0.0%)
		Strongly disagree	2 (10.0%)
5	Perceived benefits observed	Reduced postoperative complications	18 (90.0%)
		Faster recovery times (shorter hospital stays)	17 (85.0%)
		Improved patient satisfaction	17 (85.0%)
		Other (e.g. early discharge, cost)	4 (20.0%)

Regarding the perceived benefits, most believed that they reduced postoperative complications to 18 (90%), resulted in faster recovery times and shorter hospital stays 17 (85%), and improved patient satisfaction 17(85%). However, a small proportion of respondents (2; 10%) strongly disagreed with statements about the perceived benefits of ERAS. The perceived challenges and support needed during implementation are shown in Table 3. More than half of the respondents, 11 (55.0%), agreed that there were significant challenges in implementing ERAS protocols, and another 2 (10.0%) strongly agreed. The most frequently reported challenges were categorized as a lack of resources 17 (85%), limited staff training 14 (70%), resistance to change 10 (50%), and insufficient patient education 10 (50%).

**TABLE 3:** Perceived challenges and support needs during implementation among perioperative care providers (*n* = 20) in Ethiopia.

S/N	Variable	Category	*n* (%)
1	Challenges to implementation	Strongly agree	2 (10.0%)
		Agree	11 (55.0%)
		Neutral	4 (20.0%)
		Disagree	1 (5.0%)
		Strongly disagree	2 (10.0%)
2	Types of challenges experienced	Lack of resources	17 (85.0%)
		Limited staff training	14 (70.0%)
		Resistance to change	10 (50.0%)
		Insufficient patient education	10 (50.0%)
3	Training effectiveness	Strongly agree	7 (35.0%)
		Agree	9 (45.0%)
		Neutral	3 (15.0%)
4	Attendance at ERAS meetings	Always	3 (15.0%)
		Often	4 (20.0%)
		Sometimes	5 (25.0%)
		Rarely	0 (0.0%)
		Never	8 (40.0%)
5	Support needs identified	Additional training sessions	15 (75.0%)
		Increased institutional support	15 (75.0%)
		More resources (e.g. improved supply chain, infrastructure, tools, and funding for ERAS components)	13 (65.0%)
		Comprehensive patient education	13 (65.0%)
		Other support elements (e.g. peer mentorship)	3 (15.0%)

Regarding the perceived effectiveness of preparatory training, 16 (80.0%) participants either agreed or strongly agreed that it was helpful, while 3 (15.0%) were neutral. However, meeting attendance was inconsistent; with only 3 (15.0%) reported always attending, while 8 (40.0%) never attended any sessions. The most frequently requested forms of support were additional training sessions and increased institutional support, reported by 15 (75%) participants each. The majority reported needing more resources and comprehensive patient education 13 (65%) each). Peer support or mentorship was not considered a major barrier 3 (15%)).

The willingness to adopt ERAS and the perceived effectiveness of the triple intervention strategy are shown in Table 4. The majority expressed a strong willingness to adopt ERAS protocols in their surgical care 17 (85.0%)). Importantly, all respondents (20, 100%) were willing to attend future ERAS training or workshops. Key factors for permanent adoption included proven patient outcome improvements 19 (95.0%), continued education and training 19(95.0%), institutional support 18 (80.0%), and peer encouragement 12 (60.0%).

**TABLE 4:** Adoption of ERAS protocol and effectiveness of triple intervention strategy among perioperative care providers (*n* = 20) in Ethiopia.

S/N	Variables/items	Categories/options	*n* (%)
1	Willingness to adopt ERAS	Very willing	12 (60.0%)
		Willing	5 (25.0%)
		Somewhat willing	1 (5.0%)
		Neutral	2 (10.0%)
		Not willing	0 (0%)
2	Willingness to attend future training	Yes	20 (100%)
		No	0 (0%)
3	Encouraging factors for adoption	Proven improvement in patient outcomes	19 (95%)
		Continued education and training	19 (95%)
		Institutional support	18 (90%)
		Peer encouragement	12 (60%)
4	Perceived effectiveness of each strategy		
	Early feeding	Strongly agree	4 (20.0%)
		Agree	11 (55.0%)
		Neutral	3 (15.0%)
		Disagree	0 (0%)
		Strongly disagree	2 (10.0%)
	Early ambulation	Strongly agree	9 (45.0%)
		Agree	6 (30.0%)
		Neutral	2 (10.0%)
		Disagree	0 (0%)
		Strongly disagree	3 (15.0%)
	Early catheter removal	Strongly agree	9 (45.0%)
		Agree	6 (30.0%)
		Neutral	3 (15.0%)
		Disagree	0 (0%)
		Strongly disagree	2 (10.0%)
5	Participants who implemented any of the strategies	Yes	16 (80.0%)
		No	4 (20.0%)
6	Which strategies were implemented	Early initiation of postoperative feeding and drinking, early ambulation, and early removal of a urinary catheter	14 (87.5%)
		Early initiation of postoperative feeding and drinking, and early ambulation	1 (6.25%)
		Early removal of a urinary catheter	1 (6.25%)

Regarding the T‐ERAS intervention strategy, comprising early feeding, early ambulation, and early catheter removal, participants generally viewed these measures favorably. For each component, 15 (75.0%) either agreed or strongly agreed with its effectiveness, while strong disagreement was infrequent (2 or fewer, ≤ 10.0%). Among those who implemented the strategy, 16 (80.0%) reported using at least one component, and 14 (87.5%) implemented all three, as shown in Table 4. Although participants were invited to provide open‐ended feedback on their T‐ERAS implementation experience, only a small number of brief comments were submitted (*n* = 8), and these did not yield additional themes beyond the structured survey responses.

## 4 Discussions

This cross‐sectional study explored healthcare professionals' familiarity with, perceptions of, and attitudes toward ERAS and T‐ERAS in Ethiopian hospitals. The findings showed a high level of conceptual awareness (65%) and a willingness to adopt ERAS (85%), with 80% of participants implementing at least one T‐ERAS element, despite notable institutional barriers. Surgeons, anesthetists, nurses, and other perioperative staff widely recognized the benefits of ERAS, particularly in improving postoperative outcomes, reducing recovery time, and enhancing patient satisfaction. The study's relevance lies in its contextual focus: although ERAS has become standard practice in many high‐income countries, its adoption in low‐resource settings remains limited. By examining both facilitators and challenges from perioperative professionals, this study contributes critical insights to inform ERAS scale‐up efforts in Africa.

In terms of knowledge and perceived benefits, participants showed strong familiarity with ERAS protocols: 13 (65.0%) rated themselves as “very” or “extremely” familiar, and 11 (55.0%) reported prior implementation experience. The majority (80.0%) agreed or strongly agreed that ERAS improves patient outcomes. However, a small proportion of participants (10%) strongly disagreed with statements about the perceived benefits of ERAS, indicating that not all providers were uniformly convinced of its value. These perceptions are consistent with previous findings from Pakistan and France [7, 13], where healthcare providers expressed broad conceptual support for ERAS despite inconsistent adoption. The perceived benefits in this study, including reduced complications (90.0%), shorter hospital stays (85.0%), and improved satisfaction (85.0%), mirror outcomes reported in international literature [14, 15], and reaffirm the clinical relevance of ERAS. However, as highlighted by Kifle et al. (2024) [10], awareness alone does not guarantee implementation, particularly in under‐resourced health systems, underscoring the need for structured support to translate knowledge into sustained practice.

Despite strong familiarity with ERAS, consistent application remained limited. Only 3 (15.8%) participants reported always using the protocols, while 8 (42.1%) rarely did so. The main barriers reported were lack of resources (85.0%), limited staff training (70.0%), resistance to change (50.0%), and inadequate patient education (50.0%). These findings mirror implementation challenges observed even in high‐income countries, such as France and England [5, 16], and resonate with broader concerns about ERAS feasibility in low‐resource environments. Although most participants in this study were based in tertiary hospitals, the systemic constraints they reported, such as limited interdisciplinary coordination and inconsistent protocol adherence, reflect patterns seen across LMIC surgical systems. The ERAS Society's guideline for LMICs notes that whereas tertiary‐level institutions may be more suitable for initial ERAS adoption, many still lack essential infrastructure, trained personnel, and standardized perioperative workflows [17]. These observations are consistent with the support needs identified in this study, particularly the demand for institutional commitment, structured training, and enhanced patient education. Encouragingly, 16 (80.0%) participants implemented at least one of the T‐ERAS components: early feeding, ambulation, or catheter removal, and 14 (87.5%) of those applied all three. These findings suggest that while providers are knowledgeable and motivated, full implementation remains limited, highlighting the need for stronger system support and context‐specific ERAS approaches.

Translating knowledge into consistent practice, therefore, depends on broader institutional and policy‐level support. This aligns with Chong et al.’s (2022) meta‐analysis, which identified organizational facilitation, perceived value, and self‐efficacy as critical predictors of healthcare technology adoption [18]. The ASOS‐2 trial also showed that even structured perioperative interventions can fall short in LMICs when system‐level issues such as staff availability, escalation protocols, and monitoring capacity are not addressed [19]. In line with this, the ERAS Society's guidance for LMICs emphasizes that context‐specific adaptation and institutional readiness are essential for sustainable ERAS integration [17]. It has also been recommended that implementation efforts prioritize identifying which elements are most effective, understanding why they work, and evaluating them within specific settings [5].

In our study, the high willingness to attend future ERAS training (100%) and the prioritization of institutional support (75.0%) reinforce that provider motivation alone is not the limiting factor [20]. While a strong positive attitude towards the protocol was evident, with approximately 85% of participants expressing willingness to adopt ERAS principles through the T‐ERAS approach, successful implementation appears to depend more critically on local ownership. This underscores the need for locally led, adaptive implementation strategies rather than externally driven models, consistent with health policy and systems research emphasizing ownership as a central driver of evidence uptake in LMICs [21]. Importantly, as health systems in high‐income countries increasingly face similar challenges related to complexity, workforce constraints, and implementation fatigue, pragmatic, streamlined approaches such as T‐ERAS may also offer transferable lessons beyond their original contexts. This aligns with emerging evidence on bidirectional learning and innovation transfer from LMICs to high‐income settings, where locally developed, feasibility‐driven innovations can inform more sustainable care pathways globally [22]. Taken together, these findings position T‐ERAS not as a dilution of ERAS principles but as a locally grounded implementation strategy with potential relevance across diverse health system contexts.

In conclusion, despite limitations related to the study's small sample size, single‐country setting, and focus on government hospitals, these findings offer meaningful insights into the current state of ERAS awareness and adoption in Ethiopia. Perioperative professionals recognize the value of ERAS and are motivated to adopt its principles. However, persistent gaps between knowledge and routine practice reveal the need for context‐sensitive strategies, stronger institutional support, and policy‐level commitment. Scaling ERAS across the health system will require not only clinical education but also structural reform, interdisciplinary engagement, and continued evaluation. This study underscores the importance of investing in ERAS as a pathway toward safer more efficient surgical care in resource‐constrained environments.

## Author Contributions

**Fitsum Kifle:** conceptualization, methodology, formal analysis, data curation, investigation, resources, project administration, funding acquisition, writing – original draft, writing – review and editing. **Peniel Kenna:** methodology, formal analysis, data curation, investigation, writing – review and editing. **Bethelehm Muleye:** data curation, investigation, project administration. **Kokeb Desta:** data curation, investigation, resources. **Salome Maswime:** conceptualization, methodology, investigation, supervision, writing – review and editing. **Bruce Biccard:** conceptualization, methodology, formal analysis, validation, investigation, supervision, funding acquisition, resources, writing – review and editing. All authors reviewed the manuscript and approved the final version for publication.

## Ethics Statement

The study has been approved by the University of Cape Town Human Research Ethics Committee (HREC) REF 220/2024 and Arbaminch University with a protocol number of DG1445.

## Conflicts of Interest

The authors declare no conflicts of interest.

## Supporting information


Supporting Information S1

## Data Availability

The data that support the findings of this study are available on request from the corresponding author. The data are not publicly available because of privacy or ethical restrictions.
