# Supplementary material for: Healthcare Workers' Acceptance and Willingness to Implement a Pragmatic Triple‐Component Enhanced Recovery After Surgery Strategy (T‐ERAS): A Cross‐Sectional Study in Ethiopian Public Hospitals
Source: World J Surg. 2026 Mar 22;50(5):1179–86. doi: 10.1002/wjs.70319 (PMC13206391; doi:10.1002/wjs.70319)
Supplement: Supplementary file 1 — Supporting Information S1 [file WJS-50-1179-s001.pdf]

Appendix 1. ERAS protocol survey instrument (including participant information and consent form)

ERAS Survey

Consent Form information for Participation in the ERAS Protocol Survey

You
are invited to participate in a survey designed to collect data on ***healthcare workers' attitudes, beliefs, and perceptions regarding the Enhanced Recovery After Surgery (ERAS) protocols.*** The survey
aims to assess your level of knowledge about ERAS, perceived benefits, perceived challenges, and willingness to adopt ERAS practices. You will be asked to complete a survey questionnaire about your knowledge, attitudes, beliefs, and perceptions regarding ERAS protocols. The survey will take approximately 10 minutes to complete. This information will be used to improve the implementation and effectiveness of ERAS protocols. Your participation is completely voluntary, and you may withdraw at any time. Your privacy and confidentiality will be strictly protected throughout the study. Thank you for considering this opportunity to contribute to medical research.

If you have any questions, please feel free
to contact me at 0948300960 or email info@n4pcc.com,

* Indicates required question

1. Email *

____

Consent:

2. By signing this consent form, you acknowledge that you have read and understood the information provided above, and you agree to participate in this survey. *

Consent Statement:

By selecting "I Agree" below, you acknowledge that you have read and understood the information provided above, and you agree to participate in this survey.

Mark only one oval.

- Yes I Agree to participate
- No, I don't want to participate

Section 1: Demographic Information

3. 1. Are you a perioperative care provider (Surgeon, anesthetist, surgical nurse)? *

Mark only one oval.

- Yes
- No

4. 2. If yes to question 1, please specify your role: *

Mark only one oval.

- Surgeon
- Anesthetist
- Surgical Nurse
- Other: ____________________

5. 3. If "Other", specify *

____

6. 4. Name of your hospital *

____

7. 5. Type of Your Hospital *

Mark only one oval.

- Public
- Private
- Non-governmental

8. 6. Level of the hospital *

Mark only one oval.

- Primary Hospital
- Secondary Hospital
- Tertiary Hospital

9. 7. Years of your medical practice: *

____

10. 8. Gender: *

Mark only one oval.

- Male
- Female

Skip to question 11

Section 2: Knowledge about ERAS

11. 1. How familiar are you with the Enhanced Recovery After Surgery (ERAS) protocols? *

Mark only one oval.

- Not familiar
- Slightly familiar
- Moderately familiar
- Very familiar
- Extremely familiar

12. 2. Did you participate in implementing the ERAS Triple Intervention Strategy cluster conducted in your hospital? *

Mark only one oval.

- Yes
- No

13. 3. How often did you implement ERAS protocols during the trial?

Mark only one oval.

- Never
- Rarely
- Sometimes
- Often
- Always

Section 3: Perceived Benefits of ERAS

14. 1. To what extent do you agree with the following statement: "Strict adherence to ERAS protocols improve patient outcomes"? *

Mark only one oval.

- Strongly Disagree
- Diagree
- Neutral
- Agree
- Strongly Agree

15. 2. Which benefits did you observe during the trial associated with the ERAS protocol? (Check all that apply)

Check all that apply.

- [ ] Reduced postoperative complications
- [ ] Faster recovery times
- [ ] Shorter hospital stays
- [ ] Improved patient satisfaction
- [ ] Other:

16. 3. If "Other", specify: *

____

Section 4: Perceived Challenges of ERAS

17. 1. To what extent do you agree with the following statement: "There were significant challenges to implementing the proposed ERAS protocols during the trial"?

Mark only one oval.

- Strongly Disagree
- Diagree
- Neutral
- Agree
- Strongly Agree

18. 2. What challenges did you face in implementing ERAS protocols? (Check all that apply)

Check all that apply.

- Lack of resources
- Limited staff training
- Resistance to change
- Insufficient patient education
- Other:

19. 3. If "Other" specify: *

Section 5: Triple Intervention Strategy

For Question A-C: To what extent do you agree with the effectiveness of the following components of the Triple Intervention Strategy during the trial?

20. A. Starting postoperative feeding and drinking early:

Mark only one oval.

- Strongly Disagree
- Diagree
- Neutral
- Agree
- Strongly Agree

21. B. Early ambulation:

Mark only one oval.

- Strongly Disagree
- Diagree
- Neutral
- Agree
- Strongly Agree

22. C. Early removal of a urinary catheter:

Mark only one oval.

- Strongly Disagree
- Diagree
- Neutral
- Agree
- Strongly Agree

23. 2. Did you implement the Triple Intervention Strategies in your practice during the trial? *

Mark only one oval.

- ( ) Yes
- ( ) No

24. If yes, which ones? (Check all that apply)

Check all that apply.

- [ ] Starting postoperative feeding and drinking early
- [ ] Early ambulation
- [ ] Early removal of a urinary catheter

Section 6: Training and Support

25. 1. How effective was the training provided before the implementation of ERAS protocols? *

Mark only one oval.

- ( ) Strongly Disagree
- ( ) Diagree
- ( ) Neutral
- ( ) Agree
- ( ) Strongly Agree

26. 2. How often did you attend the regular meetings during the trial?

Mark only one oval.

- Never
- Rarely
- Sometimes
- Often
- Always

27. 3. What additional support would have been helpful during the trial? (Check all that apply)

Check all that apply.

- [ ] More resources
- [ ] Additional training sessions
- [ ] Increased institutional support
- [ ] More comprehensive patient education materials
- [ ] Other

28. If "Other", specify: *

____

Section 7: Willingness to Adopt ERAS Practices

29. 1. How willing are you to adopt ERAS practices permanently in your surgical care following the trial? *

Mark only one oval.

- Not Willing
- Somewhat Willing
- Neutral
- Willing
- Very Willing

30. 2. Would you be willing to participate in further training or workshops on ERAS protocols? *

Mark only one oval.

- Yes
- No

31. 3. What factors would encourage you to adopt ERAS practices permanently? (Check all that apply) *

Check all that apply.

- [ ] Proven improvement in patient outcomes
- [ ] Institutional support
- [ ] Peer encouragement
- [ ] Continued education and training
- [ ] Other

32. 4. If "Other", specify: *

____

33. Section 8: Additional Comments

34. 1. Please provide any additional comments or suggestions regarding the implementation of ERAS protocols and the Triple Intervention Strategy:

This content is neither created nor endorsed by Google.

Google Forms
